# Reusable, Recyclable, and Biodegradable Heat-Shrinkable Melt Cross-Linked Poly(butylene adipate-*co*-terephthalate)/Pulp Biocomposites for Polyvinyl Chloride Replacement

**DOI:** 10.1021/acssuschemeng.4c00012

**Published:** 2024-03-15

**Authors:** Angelica Avella, Mathieu Salse, Valentina Sessini, Rosica Mincheva, Giada Lo Re

**Affiliations:** † Department of Industrial and Materials Science, Chalmers University of Technology, Rännvägen 2A, 41258 Gothenburg, Sweden; ‡ Laboratoire MATEIS, Institut National des Sciences Appliquées Lyon, Bât. B. Pascal, Avenue Jean Capelle, 69621 Villeurbanne, France; § Department of Organic and Inorganic Chemistry, Institute of Chemical Research “Andrés M. del Río” (IQAR), Universidad de Alcalá, Campus Universitario, Alcalá de Henares, 28871 Madrid, Spain; ∥ Laboratory of Polymeric and Composite Materials, University of Mons (UMons), 7000 Mons, Belgium; ⊗ Wallenberg Wood Science Centre, Chalmers University of Technology, Kemigården 4, 41296 Gothenburg, Sweden

**Keywords:** reactive extrusion, end-of-life, heat-shrinkable films, PBAT, pulp fibers

## Abstract

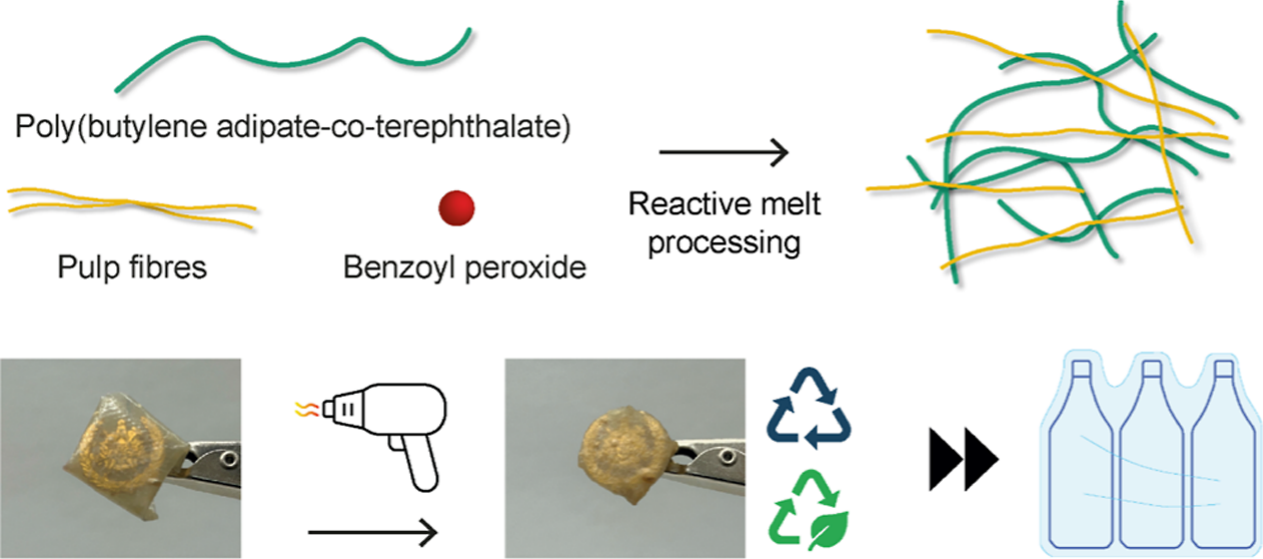

Heat-shrinkable films are widely used as disposable secondary packaging but are conventionally made from fossil-based and nonbiodegradable polyvinyl chloride or polyethylene. To lower the environmental impact of such products, this work reports the development of recyclable, biodegradable, and partially biosourced heat-shrinkable biocomposites that are cost-competitive with existing shrink wraps. Poly(butylene adipate-*co*-terephthalate), a growing biodegradable thermoplastic, was simultaneously reinforced with pulp fibers and partially cross-linked in a single-step reactive melt processing. The designed peroxide-initiated reaction led to a 55 wt % cocontinuous insoluble gel incorporating all the pulp fibers into a cross-linked polymer network. In the solid state, the cross-linked biocomposite shows 60% elongation at break with a 200% increase in Young’s modulus, while the only addition of pulp fibers stiffens and embrittles the matrix. Creep tests in the melt state indicated that the cross-linked network induces homogeneous shrinking even during the loading phase, demonstrating the potential use of the biocomposites as heat-shrinkable films. The shrinking also promotes the shape-memory of the biocomposite, which retains its dimensions after four cycles. The circularity of the materials was assessed by mechanical recycling and industrial composting, which have proven feasible end-of-life options for heat-shrinkable biocomposites.

## Abstract 2

Poly(butylene adipate-*co*-terephthalate) and pulp fibers heat-shrinkable biocomposites are produced by melt cross-linking as reusable, recyclable and biodegradable alternative to polyvinyl chloride packaging.

## Introduction

Heat-shrinkable films are a common and indispensable single-use packaging solution for the stabilization and protection of goods during sale and transportation. Usually, they are based on thermoplastic that is stretched and oriented during production and can shrink and wrap around a product upon heating. When the films are produced, cooling is used to fix the polymer chains in reversible oriented configurations, and when they are reheated, the configurations return to their random state more thermodynamically stable. This entropy-driven phenomenon allows the films to shrink upon heating.^1^ The shrink films are characterized by high puncture resistance, good shrinkage, and shrinkage stress related to certain mechanical and rheological performances.

Common applications of heat-shrinkable films include wrapping packs of bottles or cans, books, magazines, and vegetables, sealing caps, and shrinkable labels. As for most single-use plastics, heat-shrinkable films are disposable and have a short service life. Nevertheless, the most common polymers employed are fossil-based not biodegradable durables such as polyvinyl chloride (PVC) and low-density polyethylene (LDPE), cross-linked or not. PVC, in particular, is known for its negative environmental impact as it is produced from cancerogenic vinyl chloride^2^ and its mechanical recycling stream releases toxic chlorinated products from unavoidable degradation.^3^ Considering that packaging contributes to the majority of global plastic waste (≈40%) and only a small fraction of the global plastic waste is recycled (<20%),^4^ it is fundamental to develop novel sustainable materials that can replace the existing packaging. The materials sustainability includes sourcing limiting fossil fuels extraction (i.e., biobased origin), effective and nonpolluting production lines, and suitable end-of-life (from both environmental and economical viewpoints), for which mechanical recycling and biodegradation are more valuable alternatives to incineration and landfilling, and able to bring circularity for single-use plastics.^5^

Looking for alternative sourcing, poly(butylene adipate-*co*-terephthalate) (PBAT) stands out as it is a biodegradable market-available polyester with relatively easy melt processability and mechanical properties similar to PVC and LDPE.^6^ The majority of PBAT globally produced is currently applied in flexible packaging,^7^ and scientific studies have also reported the use of PBAT for heat-shrinkable films. Long et al., have produced blends of PBAT and thermoplastic starch by extrusion, followed by hot pressing and UV irradiation of the films.^8^ Thanks to the UV cross-linking, the blends displayed heat-driven shape-memory and heat-shrinkage. Pietrosanto et al., have reported heat-shrinkable blends of PBAT and poly(lactide) melt compounded with a chain extender.^9^ The blends were shaped by film-blowing, and the heat-shrinking was achieved mainly in the machine direction, as the films were unidirectionally oriented. Yu et al., have shown that pure PBAT can heat shrink and it displays shape-memory.^10^ They attributed this phenomenon to the amorphous and crystalline phases of the polyester that can be respectively considered the stationary and reversible phases that originate the shape-memory.

In packaging applications, good shrinkage is required, together with certain mechanical and rheological performances. To improve the mechanical and rheological properties of PBAT, it can be blended with natural fillers into biobased composites.^11−13^ In particular, cellulose is a good candidate as a reinforcing agent considering its abundance, low cost, renewability, biodegradability, and role as a reinforcing agent.^14,15^ Other works have blended PBAT with nanosized cellulose^16^ and found challenging its dispersion into the polymer matrix due to immiscibility allowing the large surface area of nanocellulose and high amount of hydroxyl groups which promote interparticle bonding, upon drying and during melt mixing.^17^ Therefore, cellulose modification is often needed to improve the mechanical properties of cellulose/PBAT biocomposites.^11^

Aiming for effective and green production, reactive melt processing (REx) is of interest, as it (i) combines materials manufacturing with simultaneous chemical reactions, (ii) does not require solvents, and (iii) is industrially relevant and energy-effective. Therefore, REx results in a sustainable technique for easy industrial uptake and has already been used in cellulose-containing composites.^18,19^

Free-radical cross-linking boosted by water during reactive extrusion was demonstrated to improve the thermomechanical and rheological properties of poly(ε-caprolactone) (PCL) and its biocomposites with cellulose nanocrystals (CNC).^18,20^ The formation of a cocontinuous cross-linked network in the biocomposites allowed them to shrink homogeneously when heated. When the biocomposites are compression molded, the cooling under pressure freezes the cross-linked chains in a stretched state and when heated the chains return to their high entropy coiled state, leading to shrinkage. Compared to PCL and CNC, PBAT and pulp fibers are significantly cheaper^21^ and commercially available^7^ thus their use favors an industrial upscaling. Pulp fibers are readily available from the pulp and paper industry without the need for the extraction required for nanocelluloses. They can provide better reinforcement and rheological^22,23^ and possibly help in achieving the functional requirements for sustainable and competitive heat-shrinkable packaging. Pulp fibers are also more thermally stable than CNC,^24^ which can prevent excessive degradation during mechanical recycling of pulp-based composites.

The present work, therefore, aims to investigate the cross-linking via reactive melt processing as a method for inducing heat-shrinking in biocomposites of PBAT and pulp fibers and analyze their performance and their end-of-life options as sustainable alternatives to LDPE or PVC shrink wraps. The designed cross-linked materials displayed heat-shrinking above PBAT melting temperature and showed shape-memory after four loading cycles. Moreover, after their use, mechanical recycling and industrial compositing were demonstrated as feasible end-of-life options, paving the way toward circularity.

## Experimental Section

### Materials

PBAT was purchased from Jinhui ZhaoLong High Technology Co. Ltd. (China), with a declared density of 1.26 g/cm^3^ and a melt flow rate ≤ 5 g/10 min (ISO 1133) at 190 °C and 2.16 kg. The pulp fibers were provided by Nordic Paper Säffle AB (Sweden) and are a never-dried (wet pulp directly collected in the production line) bleached mixture of 80% spruce sulphite and 20% spruce sulfate pulp. The moisture content was 73 wt % and the fibers’ composition is approximately 80 wt % cellulose, 18 wt % hemicellulose and 2 wt % lignin. The dimensions of the fibers measured by Kajaani FS300 fiber analyzer were: average length 1.36 mm and width and 24.4 μm (aspect ratio of ≈55). Benzoyl peroxide (BPO) was purchased from Sigma-Aldrich AB (Sweden) and was used without further purification. Dichloromethane (DCM) was purchased from VWR International AB (Sweden) with a purity higher than 99.5%. Heat-shrinkable sleeves in polyvinyl chloride were provided by Åre Skidfabrik (Sweden) and produced by Xinjiang Huanghe Packing Co. Ltd. (China).

### Methods

#### Reactive Melt Processing

The materials produced by reactive melt processing are defined in Table 1 together with their composition. To fabricate X-P-PBAT, PBAT pellets were manually mixed with never-dried pulp fibers (15 vol % in the dry state) and 1.6 phr of benzoyl peroxide (weighted in comparison to the PBAT mass). The mixture was melt-processed in an internal mixer Brabender AEV 330 (50 cm^3^) with counter-rotating screws at 130 °C. The process was carried out for a total of 12 min, feeding for 2 min at 30 rpm, then 10 min at 60 rpm. The processing time took into account that the BPO half-life at 130 °C is 1 min.^25^ The same method was followed for a PBAT-pulp biocomposite (P-PBAT), a reference of PBAT with deionized water in the same amount present in 15 vol % of never-dried pulp fibers (PBAT_H_2_O), and cross-linked PBAT in the presence of water (X-PBAT).

**Table 1:** Contents of PBAT, Benzoyl Peroxide, Dry Pulp, and Water in the Neat and Produced Materials, with Relative Acronyms Used in this Study

material	PBAT [vol %]	peroxide [phr]^a^	dry pulp [vol %]	H_2_O [phr]^a^
PBAT	100			
PBAT-H_2_O	100			44
X-PBAT	100	1.6		44
P-PBAT	85		15	44
X-P-PBAT	85	1.6	15	44

a Measured in relation to PBAT weight.

All of the materials were shaped into squared films of 1 mm thickness using a compression molder Buscher-Guyer KHL 100 at 130 °C for 3 min at 40 bar and 1 min at 500 bar, followed by quenching to room temperature under pressure. For demonstration of heat-shrinkage and for disintegration tests, the thickness was reduced to 0.1 mm.

#### Mechanical Recycling

The compression-molded films of the cross-linked materials (X-PBAT and X-P-PBAT) were shredded and processed in an Xplore microcompounder MC15HT to assess their mechanical recyclability. Fourteen g of each material were extruded at 130 °C, at 30 rpm during a 5 min feeding and at 60 rpm during a 5 min processing. The extrudates were injection molded (Xplore IM12) into Dumbbell-shaped specimens, with a barrel temperature of 140 °C (mold at 25 °C), following an injection program of 5 s at 280 bar and holding for 10 s at 420 bar. The recycled samples are denoted as Re-X-P-PBAT (recycled cross-linked biocomposite) and Re-X-PBAT (recycled cross-linked PBAT).

#### Disintegration under Composting Conditions

The disintegration test under aerobic composting conditions mediated by thermophilic bacteria was performed at a laboratory scale following the ISO 20200:2015 standard. The materials were cut into square-shaped samples (25 mm × 25 mm × 0.1 mm), weighted and they were contained in a textile mesh to allow their easy removal after the composting test, but also allowing the access of moisture and microorganisms. They were buried at 4–6 cm depth in perforated plastic boxes containing a solid synthetic wet waste (10% of compost, 30% rabbit food, 10% starch, 5% sugar, 4% corn oil, 1% urea, 40% sawdust, and approximately 50 wt % of water content) and were incubated at aerobic conditions (58 °C). The aerobic conditions were guaranteed by periodic mixing of the solid synthetic wet waste. One sample of each formulation was recovered from the disintegration container at different times (7, 21, 28, 39, 56, and 90 days). The film samples were then cleaned with distilled water, dried in an oven at 37 °C for 24 h, and reweighed. The disintegration degree was calculated by normalizing the sample weight at the different days of incubation to the initial weight.

#### Characterization Methods

The length and width of the neat pulp fibers were determined as the mean values by using a Kajaani FS300 fiber analyzer (Metso Automation, Finland) according to the Tappi T271 18 standard. The found average length and width were 1.36 mm and 24.4 μm, respectively (aspect ratio of ≈55) and the counted fines were 11.9%.

Soxhlet extraction was carried out on 5 g samples of each material (except neat PBAT) in 500 mL of DCM for 72 h using glass fiber thimbles (Whatman 603G, VWR). The extracted insoluble fractions were then dried into Petri dishes at room temperature for 48 h and then weighted to measure the gel content according to the eq 1
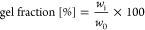
1where *w*_i_ is the dry weight of the insoluble fraction and *w*_0_ is the initial weight of the sample. To separate possible pulp physically entrapped in the gel, the extracted insoluble fraction of X-P-PBAT was redispersed in DCM and centrifuged in a Heraeus Labofuge 200 Centrifuge (Thermo Scientific) at 5000 rpm for 5 min.

Attenuated Total Reflectance Fourier-Transform Infrared Spectroscopy (ATR-FTIR) was performed with a PerkinElmer FT-IR Spectrometer Frontier in ATR mode. Twenty scans were acquired from 4000 to 400 cm^–1^ with a resolution of 4 cm^–1^. All data were recorded using the PerkinElmer Spectrum software.

Size-exclusion chromatography (SEC) was carried out in CHCl_3_ at 30 °C by using an Agilent (Diegem) liquid chromatograph. Polystyrene (PS) standards were used for calibration. The instrument was equipped with an Agilent degasser, an isocratic HPLC pump (flow rate = 1 mL min^–1^), an Agilent autosampler (loop volume = 100 μL; solution concentration = 2 mg ml^–1^), an Agilent-DRI refractive index detector, and three columns [a PL gel 5 μm guard column and two PL gel Mixed-B 5 μm columns, linear columns for separation of molecular weight (PS) ranging from 200 to 4 × 10^5^ g mol^–1^].

The injection-molded samples were cryo-fractured in liquid nitrogen, and the surfaces were sputtered with gold in vacuum for 1 min at 10 mA. The samples were then investigated with an Ultra 55 FEG Scanning Electron Microscopy (SEM) (Zeiss Sigma) under an accelerating voltage of 3 kV.

Creep and shape-memory properties were measured with a DMA Q800 (TA Instruments) apparatus in tension-film mode on rectangular bars (25 × 5 × 1 mm^3^). The bars were cut from compression-molded films and conditioned for 48 h at 23 °C and 53% relative humidity. Creep tests in the melt state were performed isothermally at 160 °C under the constant stress of 1 kPa, with a 30 min displacement time and a 30 min recovery time. Before displacement, the samples were allowed to equilibrate at 160 °C for 2 min.

To test the shape-memory, the samples were initially heated to 150 °C and equilibrated for 60 min to relax the material. After the isothermal step, indicating the beginning of the cycle, the length of the sample was measured, and a stress was applied until reaching 20% of strain. This value of strain was selected to highlight the shape-memory effect. Then, the stress was kept constant during cooling to 70 °C and further isotherm for 15 min, after which the stress was removed. The sample was left unloaded for 5 min at 70 °C and then reheated to 150 °C at a rate of 3 °C/min for 40 min. Five cycles were performed and the shape-recovery (*R*_r_) and the shape-fixity (*R*_f_) ratios were calculated for each cycle, according to eqs 2 and 3, respectively.^26,27^ The *R*_r_ quantifies the ability of the material to recover its shape and is calculated as the ratio between the strain recovered and the maximum strain (ε_m_) reached during the cycle
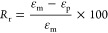
2where ε_p_ is the strain in the recovered state. The *R*_f_ quantifies the ability of the material to fix the temporary shape and it is calculated as the ratio between the strain after the stress was removed (ε_u_) and the maximum strain (ε_m_)
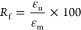
3

Dynamic rheological measurements were carried out with an Anton Paar MCR 702 rheometer in single-drive mode with a parallel plate geometry (15 mm ⌀). Disks (16 mm ⌀) were cut from compression-molded films and were conditioned for 48 h at 23 °C and 53% relative humidity. The disks were tested isothermally at 160 °C and a gap of 1 mm after removal of melt material exceeding the selected geometry. Frequency sweep tests were carried out in an angular frequency range from 0.1 to 200 rad s^–1^ at an applied strain of 1% within the linear region.

The tensile properties were tested on Dumbbell’s specimens cut from compression-molded sheets, with an effective length of 25 mm, thickness of 1 mm, and width of 4 mm. The samples were conditioned for 48 h at 23 °C and 53% relative humidity before testing. At least 5 specimens for each material were tested with a Zwick/Z2.5 tensile instrument (ZwickRoell) equipped with a load cell of 2 kN at a crosshead speed of 6 mm/min. Dumbbell’s specimens were also cut from the PVC heat-shrinkable sleeves and tested under the same conditions.

The thermal stability was studied by thermogravimetric analysis (TGA) with a TGA/DSC 3+ Star system (Mettler Toledo). Approximately 5 mg samples were preheated in alumina crucibles from room temperature to 70 °C, with a 15 min isothermal segment for residual moisture evaporation. Then the samples were heated to 550 °C at a heating rate of 5 °C min^–1^, under N_2_ 50 mL min^–1^ flow. The onset temperature of degradation (*T*_5%_) was identified as the temperature at 5% weight loss. The temperature of degradation (*T*_d_) was reported as the peak temperature of the first derivative (DTG). Char residue was estimated as the final weight % at 550 °C.

Thermal transitions and crystallinity were evaluated by differential scanning calorimetry (DSC) with a Mettler Toledo DSC 2 calorimeter equipped with an HSS7 sensor and a TC-125MT intercooler. The endotherms were recorded following a heating/cooling/heating temperature profile from −80 to 200 °C, at a heating rate of 10 °C min^–1^, under N_2_ 50 mL min^–1^ flow. The melting temperature (*T*_m_) was detected as the peak temperature of the melting transition in the second heating scan, while the glass transition temperature (*T*_g_) was at the flex point of the transition step. Crystallization temperature (*T*_c_) was evaluated as the peak temperature of the crystallization transition in the cooling scan. The degree of crystallinity (χ_c_) was calculated according to eq 4
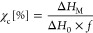
4where Δ*H*_M_ is the specific melting enthalpy, Δ*H*_0_ is the melting enthalpy of 100% crystalline PBAT (114 J/g^28^) and *f* is the weight fraction of PBAT.

## Results and Discussion

### Reactive Melt Processing

**Figure 1:**
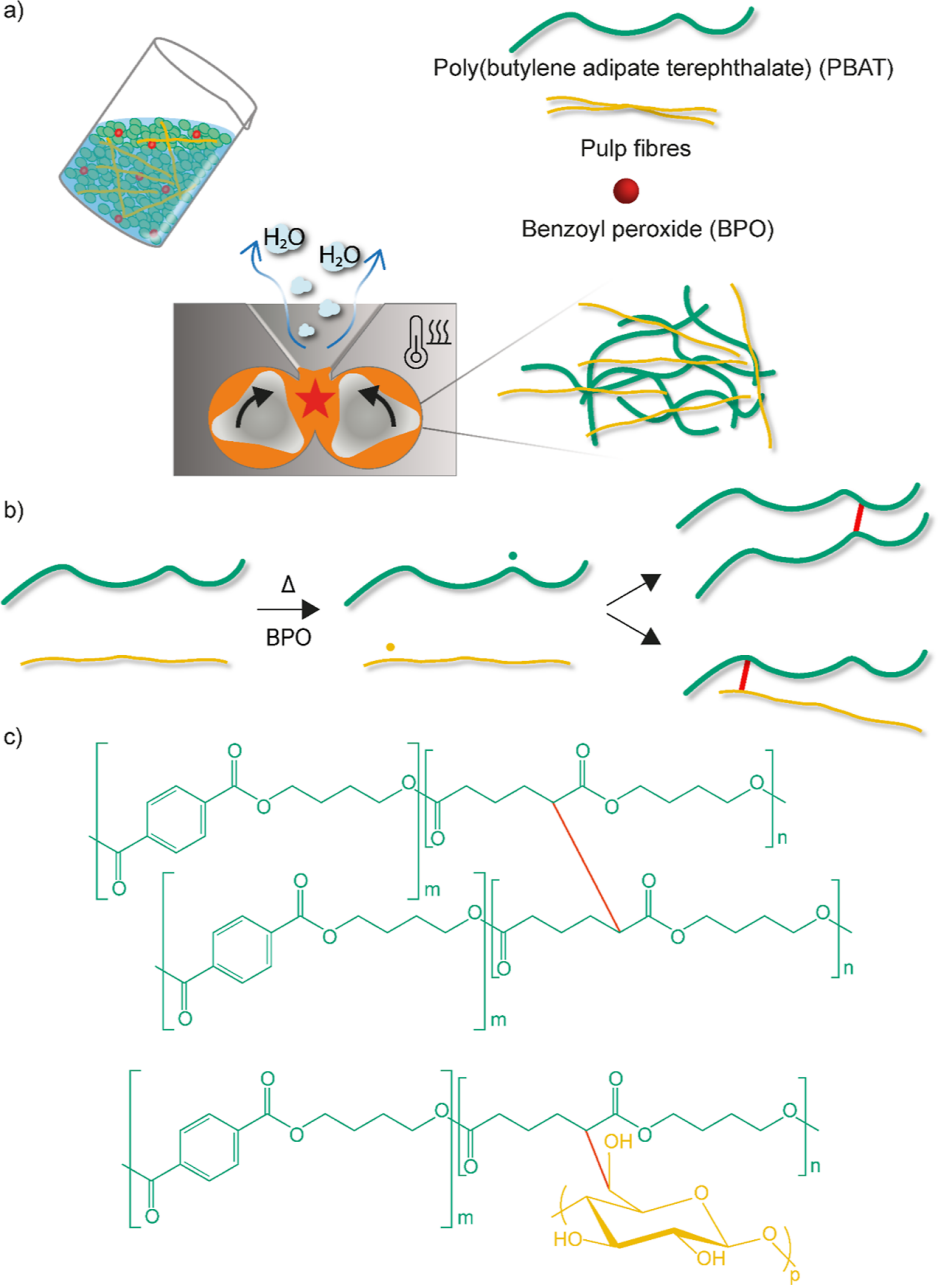
(a) Scheme of free-radical cross-linking during reactive melt processing of never-dried pulp fibers with PBAT. (b) Reaction scheme proposing the formation of radicals on the PBAT chain and cellulose backbone and their covalent bonding and covalent bonding between PBAT chains. (c) Chemical structures of cross-linking between PBAT chains and PBAT-pulp fibers.

To produce a circular heat-shrinkable biocomposite, PBAT and 15 vol % of never-dried pulp fibers (X-P-PBAT) were cross-linked during reactive melt processing in an internal mixer (Figure 1). The fibers were fed in the never-dried state to reduce their agglomeration during melt processing and to exploit the water for boosting the radical reactions.^18,20^

**Figure 2:**
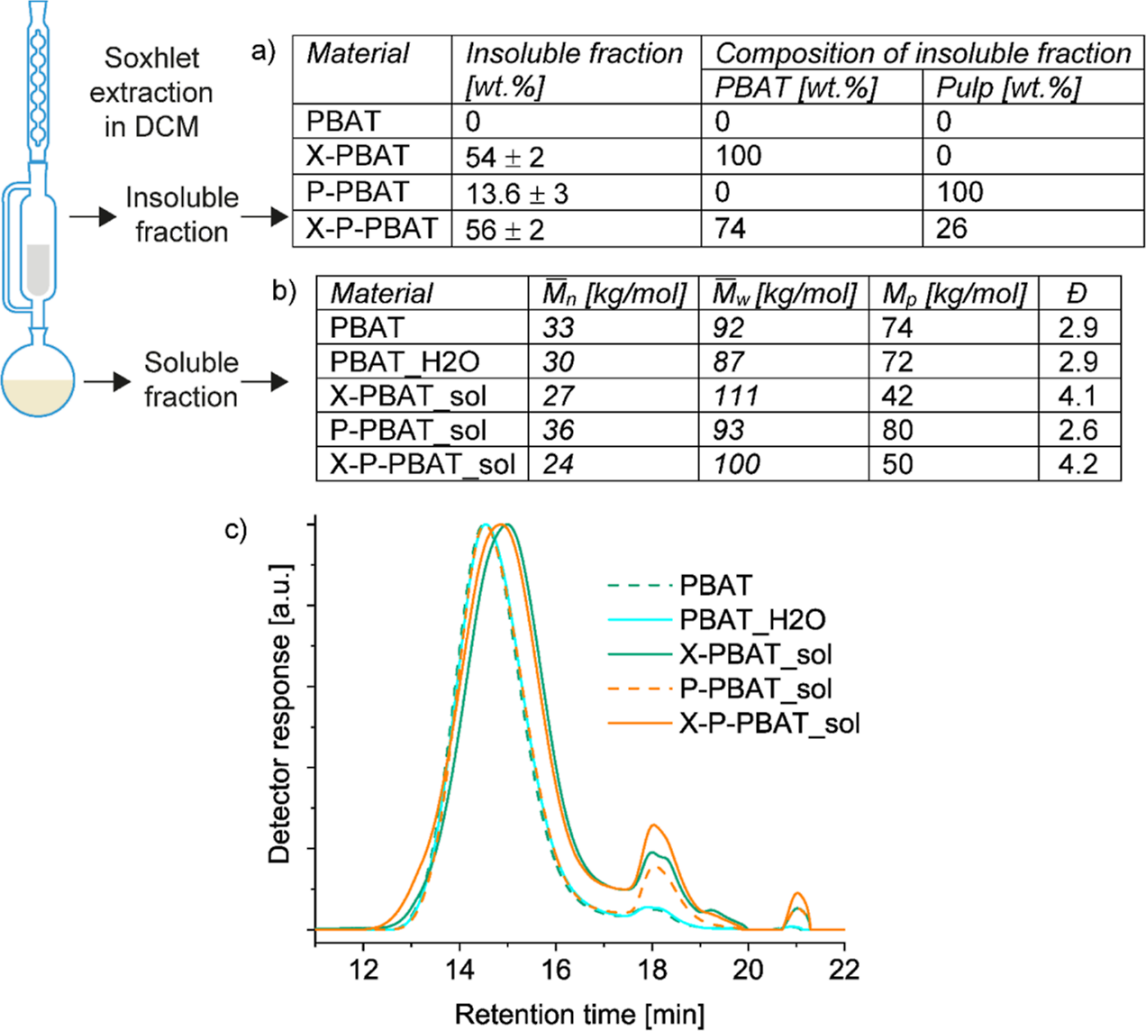
Scheme of Soxhlet extraction in dichloromethane with (a) table reporting amount and composition of the insoluble fractions; (b) table with the number () and weight () average molecular weights, peak molecular weight (*M*_p_) and polydispersity (*D̵*) of the soluble fractions measured by size-exclusion chromatography and (c) the corresponding chromatographs detected in chloroform.

Three references were processed to explore the influence of the fibers and cross-linking on the biocomposite properties: PBAT in the presence of water, PBAT cross-linked with the same amount of peroxide in the presence of water (X-PBAT) and PBAT/pulp biocomposite without peroxide (P-PBAT). PBAT was first processed with the equivalent amount of water as present in the never-dried fibers to study the extent of possible hydrolysis occurring during melt processing. Water was completely evaporated during the melt processing, as confirmed by TGA analysis in which the percentage of mass loss during the isotherm at 70 °C is negligible (Figure S4). No significant change (<5%) in PBAT average molecular weight () or polymer dispersity (D̵) measured by SEC occurred after processing with the (Figure 2b,c), indicating negligible hydrolysis. To enable melt processing (compounding and compression molding) and limit the degree of cross-linking below a full thermoset system, a selected amount of peroxide (1.6 phr of PBAT) was chosen to control the amount of cross-linking.

To verify the outcome of the reaction, the produced materials were subjected to a Soxhlet extraction from dichloromethane, in which neat PBAT is fully soluble, while pulp and any cross-linked material are not. This enabled the extraction of any free PBAT chains, the gravimetric quantification of the cross-linking degree, and the analysis of the soluble and insoluble fractions of the materials. When the peroxide decomposes with temperature, radicals are formed and propagated on PBAT chains and pulp backbone (Figure 1b).^29,30^ The radicals on PBAT chains can preferentially form on aliphatic carbons^31−33^ while some studies hypothesize radical formation on the aromatic ring.^34,35^ PBAT radicals can further react forming covalent bonds with other PBAT chains or pulp, or can break the chains by β-scission.^31^ The intermolecular bonds increase the molecular weight (branching) and finally lead to an insoluble network (cross-linking).

The insoluble fraction of the biocomposite prepared by compounding, without the addition of peroxide, resulted in 13.6 wt % (≈15 vol %), a value similar to the percentage of added pulp, thus suggesting that Soxhlet extraction served merely to separate the fibers from the PBAT. The insoluble fraction of the cross-linked biocomposite was around 55 wt %, similar to the one of neat PBAT cross-linked with the same amount of peroxide (Figure 2a). The similarity is ascribed to the same reactivity of benzoyl peroxide in both systems. In the cross-linked biocomposite, radicals could have formed both on PBAT chains and on pulp fibers (Figure 1b),^29^ leading to an insoluble fraction containing PBAT and fibers (26 wt % of the insoluble fraction of X-P-PBAT, corresponding to the 15 vol % of fibers present in the biocomposite). Surprisingly, all of the fibers initially blended in the biocomposite were irreversibly incorporated in the insoluble fraction. To further confirm this result, the insoluble fraction after Soxhlet was centrifuged to separate possible pulp physically entrapped in the gel, and no precipitate was collected, indicating that all the fibers were grafted to the PBAT network.

The recovered insoluble fraction was then analyzed by FTIR which confirmed the predominant presence of PBAT covering the pulp fibers (Figure S1 in the Supporting Information). A shoulder at 1061 cm^–1^ is observed in the insoluble fraction of X-P-PBAT and can be ascribed to the cellulose backbone,^36,37^ slightly shifted to a higher wavelength compared to the pulp spectrum. On the contrary, in the FTIR spectrum of the insoluble fraction of the unreacted P-PBAT biocomposite, only typical signals of pulp fibers, not soluble in dichloromethane, were detected.

The molecular characteristics of the soluble fractions, although not representative of the entire sample, were studied by SEC to evaluate the effect of the reaction on PBAT (Figure 2b,c). The elution curves showed a reduction of PBAT molar mass and larger polydispersity after cross-linking. The lower molecular weight can be a consequence of both the promotion of branched chains to the cross-linked insoluble network and β-scission. A small shoulder at higher molar mass is also detected, confirming the branched structure (Figure 2c).

### Biocomposites Properties

**Figure 3:**
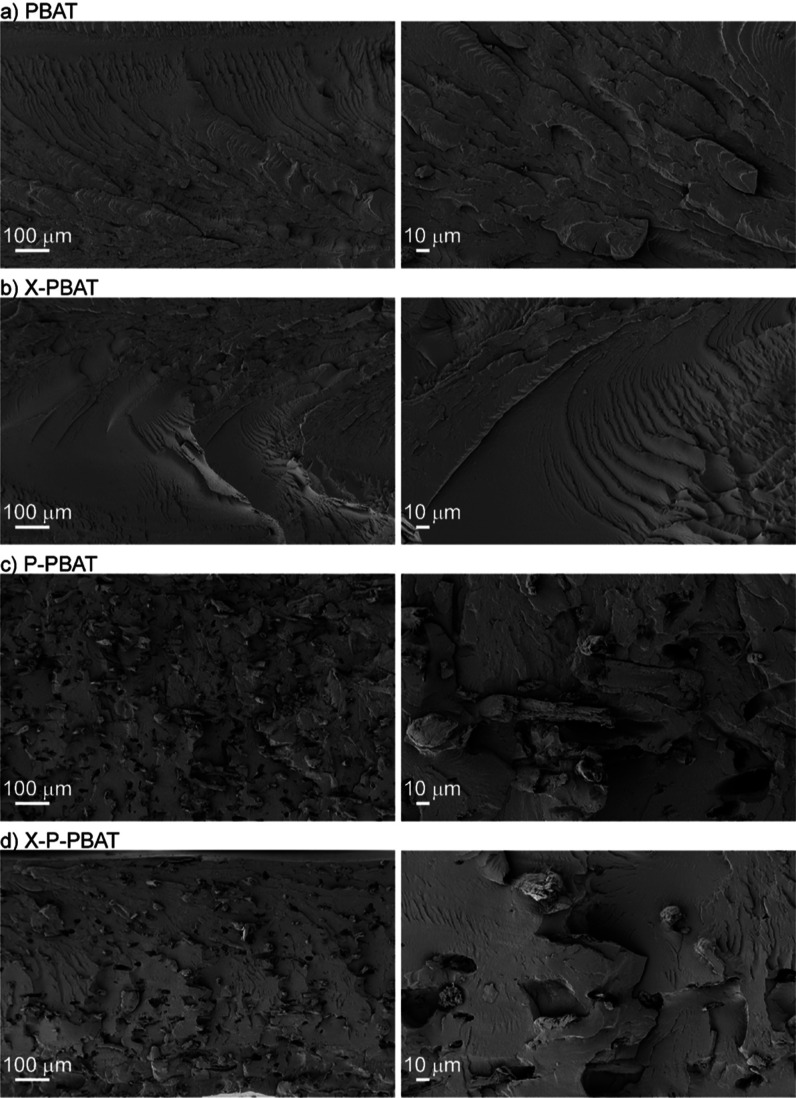
Representative scanning electron microscopy micrographs of cryo-fractured surfaces from compression molded films of (a) PBAT, (b) X-PBAT, (c) P-PBAT and (d) X-P-PBAT at two different magnifications (scale bars 100 and 10 μm).

The as-processed materials were characterized by SEM to observe how the reactive processing influences their morphology and if cross-linking has an effect on the pulp dispersion and interaction with PBAT. The micrographs of PBAT cryo-fracture show a typical brittle surface fracture of a ductile polymer in its glassy state, and it is not influenced by cross-linking (Figure 3a,b). Few micrometrical fibers in bundles are detected in both biocomposites (Figure 3c,d, at both magnifications) but the majority of cellulosic fibers appears fibrillated,^38−40^ as a consequence of shear forces induced during melt processing and the beneficial wet-feeding approach, which prevents fibers agglomeration. The fracture surface of the P-PBAT biocomposite is characterized by numerous voids, which are correlated to the pull-out of the fibers (Figure 3c), indicating poor adhesion at the interface pulp/PBAT. In the cross-linked X-P-PBAT biocomposite (Figure 3d), the number and size of the voids decrease, indicating that cross-linking reduces the agglomeration of the fibers and improves the interface with PBAT. The discrepancy between the number of voids, aggregates, and pull-outs can be ascribed only to the formation of pulp/PBAT hybrids during cross-linking.

**Figure 4:**
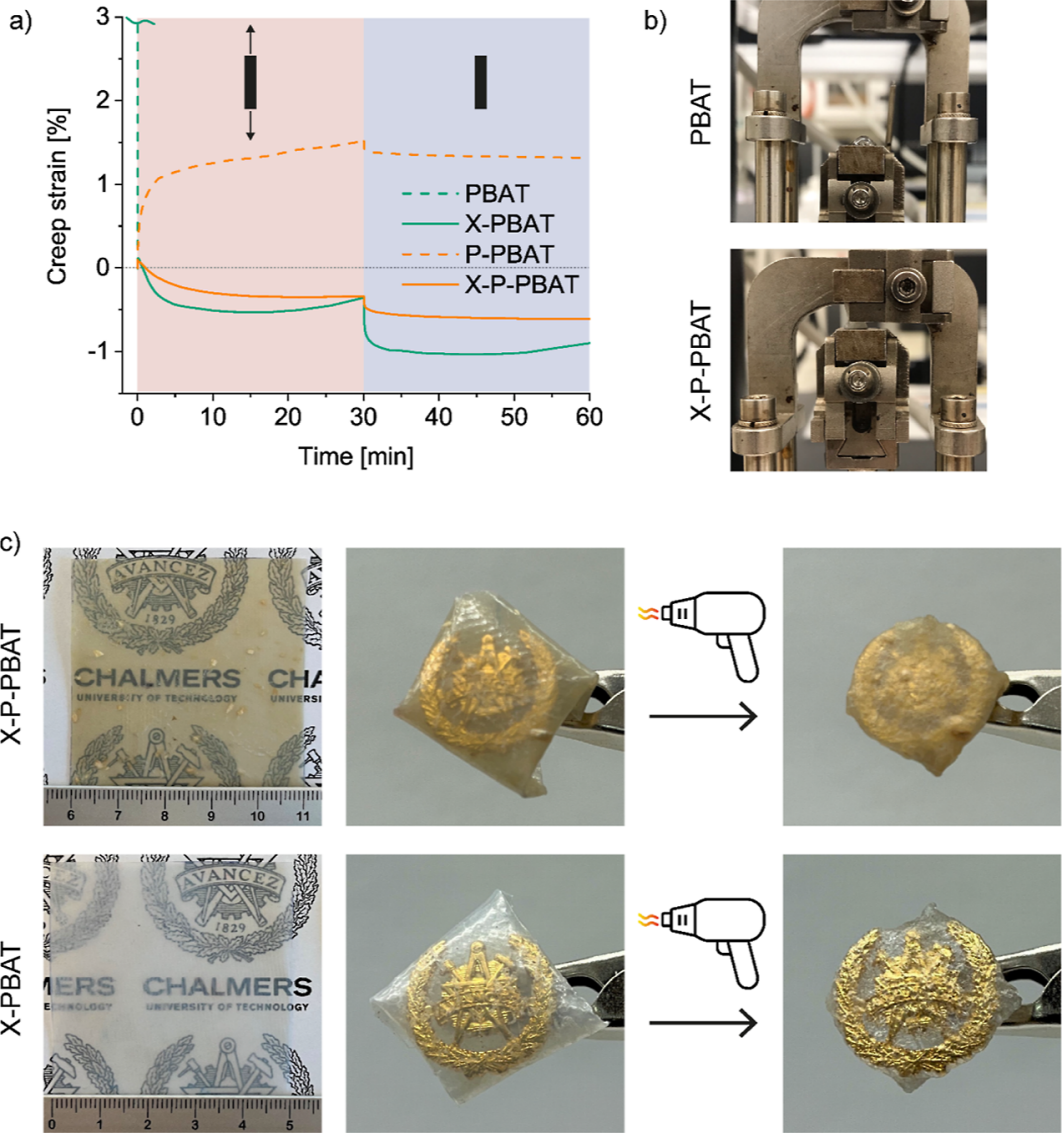
(a) Creep curves recorded at 160 °C under a load of 1 kPa for 30 min followed by 30 min recovery. (b) Photographs of PBAT and X-P-PBAT specimens after the creep test. (c) Photographs of X-P-PBAT and X-PBAT films (70–100 μm) wrapped around a pin and after being heated with a heat gun for 15 s. The logo in the photographs is used with permission from Chalmers University of Technology.

Creep tests at 160 °C were carried out to verify whether the materials shrink when heated above the PBAT melting temperature (123 °C). Due to its melting, PBAT quickly strains and breaks when a tensile load (1 kPa) is applied (Figure 4a,b). The only addition of pulp avoids PBAT fracture, and it limits the creep strain to less than 2% after half an hour of loading; however, the biocomposite is not able to recover the deformation. This result indicates a large elasticity in the melt state provided by the fibers. Both X-PBAT and X-P-PBAT uniformly shrink when exposed to tensile load and high temperature, i.e., the creep strain is negative. The uniformity of the shrinkage indicates a cocontinuity of the cross-linked network in the materials. During compression molding, the network is fixed into a stretched state at low entropy and at high temperature it coils to a high entropy state.^18^ The creep curve of the cross-linked biocomposite reaches a plateau during both load and recovery steps. Instead, the cross-linked PBAT first shrinks and then relaxes, i.e., the strain increases with time during both load and recovery. This difference in the cross-linked materials is ascribed to the elasticity provided by the pulp fibers, which limits the shrinkage of X-P-PBAT compared to X-PBAT but ensures higher dimensional stability.

The heat-shrinking observed in the cross-linked materials was also tested by simulating their potential application as shrink films. A pin was wrapped with a thin film (0.1 mm) of X-PBAT or X-P-PBAT and it was heated with a heat gun until the films shrank to the dimensions of the pin (Figure 4c). The shrinkage was achieved in around 15 s, demonstrating the feasibility of both cross-linked materials to be applied as alternatives to currently commercially available shrink films. If the films are left free to shrink under no stress in an oven at 160 °C, their deformation is isotropic and the shrinkage is 40 and 60% of their initial shape for X-P-PBAT and X-PBAT, respectively (Figure S2 in Supporting Information). These values are in similar ranges of oriented heat-shrinkable PVC and PE films, indicating a comparable functional performance of the biocomposites.^41,42^

**Figure 5:**
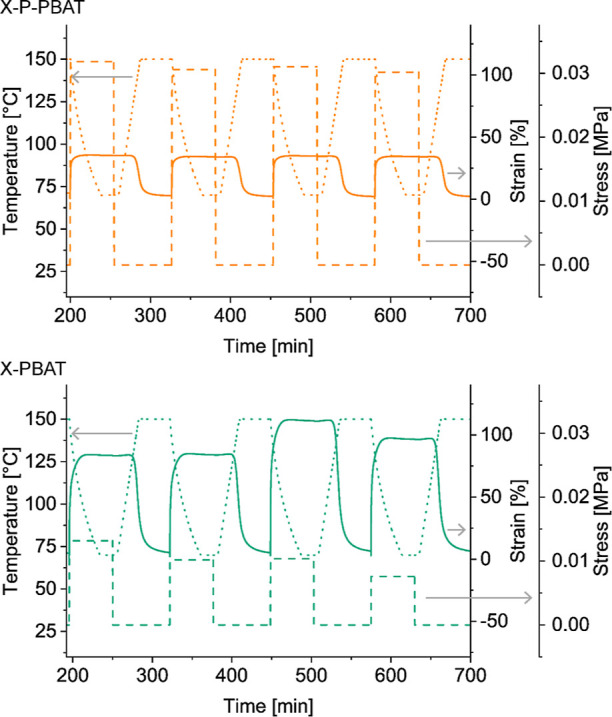
Temperature (dotted line), stress (dashed line), and strain (continuous line) as functions of time during shape-memory tests of X-P-PBAT and X-PBAT. The stabilizing isotherm and first cycle are omitted.

The heat-shrinking implies a shape-memory phenomenon, as suggested by the creep test during which the cross-linked materials can retain their shape at high temperatures and under a load. To further explore this feature, the shape-memory was tested to understand if the shape of the films can be restored after multiple deformations. Shape-memory polymers are a class of smart materials that recover a defined shape under a trigger,^43^ in this case temperature. Therefore, the samples were heated (150 °C) and stretched to a set deformation (20%), and then, they were cooled (70 °C) while keeping the stress constant to fix the temporary shape. The stress was then removed, and the samples were free to recover their shape by reheating. Figure 5 reports the temperature, stress, and strain for X-P-PBAT and X-PBAT during four shape-memory cycles. Over the cycles X-P-PBAT strains less than X-PBAT (≈35 and 90% respectively), given the elasticity provided by the pulp fibers in the network. The level of stress and maximum strain of X-P-PBAT remain similar during the cycles, indicating a more homogeneous response of the cross-linked network in the biocomposite compared to one in X-PBAT, which shows more oscillating values of stress and strain. This variation in the strain values observed for X-PBAT can be ascribed to the lower elasticity of the cross-linked polymer without the pulp fibers, and it is consistent with the higher shrinkage and relaxation observed in the creep analysis (Figure 4a). The shape-memory behavior was quantified by the shape-recovery (*R*_r_) and shape-fixity (*R*_f_) ratios, which measure the ability of the sample to recover its shape or to retain the shape in its fixed state (Table S1 in the Supporting Information). Both materials show high shape-recovery (*R*_r_ = 94%) and shape-fixity (*R*_f_ = 100 and 99% for X-P-PBAT and X-PBAT, respectively), confirming strong shape-memory behavior. This behavior indicates that even after stretching, the films could go back to their pristine shape and be stretched again for at least the assessed 4 cycles, i.e., reused multiple times.

**Figure 6:**
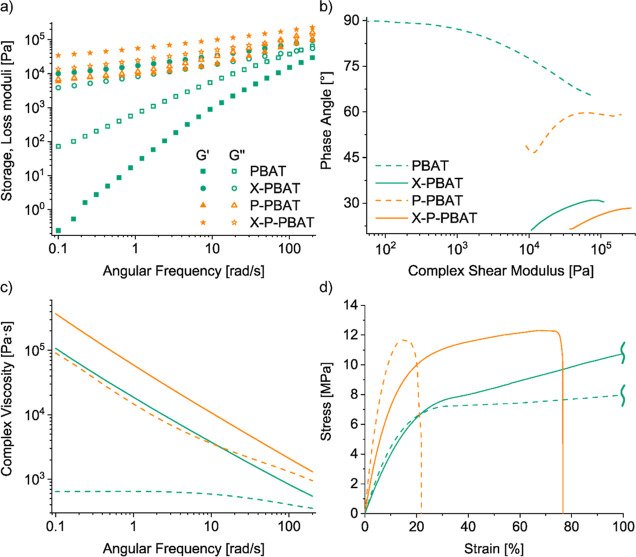
Dynamic rheological frequency sweeps at 160 °C: (a) storage and loss moduli; (b) van Gurp–Palmen plot showing phase angles as a function of complex shear modulus; (c) complex viscosity. (d) Representative tensile curves at room temperature.

Rotational rheology at 160 °C was carried out to understand the influence of the pulp fibers and cross-linking on the rheological behavior of PBAT, in relation to its molecular structure. The loss modulus of the neat matrix is higher than the storage modulus in the entire frequency range tested, indicating a prevalent liquid-like behavior of the melt (Figure 6a). The addition of pulp fibers increases the moduli and reduces the gap between the loss and storage moduli, especially in the low-frequency region, confirming a higher elasticity of the biocomposite in the melt state. The effect of the pulp on the rheological properties of PBAT is greater than the neat effect of cross-linking. The not-reacted composite has overlapping *G*′ and *G*″ up to 1–2 rad/s and increasing the frequency the dissipative part prevails, indicating a disruption of the pulp fibers network. In contrast, the cross-linked biocomposite shows a prevalent solid-like behavior in all the frequency range observed (*G*′ > *G*″), with over three times higher *G*′ values than the not-reacted counterpart. The cross-linked biocomposite displays the largest moduli, given by both the elastic contribution of the pulp and the increased PBAT molecular weight by cross-linking. This result demonstrates the stable contribution to the elastic behavior of the permanent hybrid network under the tested conditions.

The van Gurp–Palmen plot (Figure 6b) displays the phase angles as a function of the absolute value of the complex shear modulus, where phase angles below 45° indicate predominant melt elasticity.^44^ PBAT liquid-like behavior is confirmed by angles above 60°, while the elastic cross-linked materials have phase angles below 30°. The complex viscosity of PBAT follows a linear Newtonian behavior until 10 rad/s, followed by shear thinning (Figure 6c). The addition of pulp increases the complex melt viscosity, and anticipates the shear thinning because of its solid structure and its networking disruption at increasing frequencies.^17^ Also the only cross-linking induces shear thinning over the entire frequency range and increases the viscosity of PBAT as a consequence of the larger molecular weight and chain entanglement.^31,45^ The two contributions are combined in X-P-PBAT, which is characterized by the largest complex viscosities over the entire range of frequencies.

The mechanical properties of the materials were evaluated by tensile tests at room temperature. Cross-linking slightly reduces the mechanical properties of PBAT, while pulp increases the stiffness of the matrix by 340% but significantly reduces its deformability (Figure 6d and Table 2). The cross-linked biocomposite shows a 200% increase in PBAT Young’s modulus and retains some deformability (≈80%), almost 4 times the values registered for the P-PBAT biocomposites. This result is consistent with the observed morphology (Figure 3c,d), indicating that cross-linking reduces the pulp agglomeration and improves its dispersion thanks to an increased PBAT/pulp interaction.

**Table 2:** Tensile Properties of the as-Processed and Recycled Materials Measured by a Tensile Test at Room Temperature with a Crosshead Speed of 6 mm/min

material	Young’s modulus [MPa]	tensile strength [MPa]	elongation at break [%]
PBAT	52 ± 2	22 ± 3	1098 ± 212
X-PBAT	46 ± 1	14 ± 1	180 ± 35
P-PBAT	177 ± 11	12 ± 1	21 ± 3
X-P-PBAT	103 ± 8	12 ± 1	64 ± 14
Re-X-PBAT	45 ± 2	15 ± 1	163 ± 21
Re-X-P-PBAT	130 ± 2	17 ± 1	54 ± 8

Neat PBAT has low crystallinity (≈10% measured by DSC), and cross-linking and pulp do not significantly alter this value (Figure S3 and Table S2 in Supporting Information), thus a nucleating effect can be excluded as a possible reason for the recorded increase in stiffness in both the biocomposites. The mechanism of reinforcement can be found in the stress transfer of the load to the more rigid pulp fibers, further improved in the cross-linked biocomposites by the improved pulp/PBAT interface, pulp dispersion, and change of PBAT molecular structure. The crystallization temperature during cooling is increased by both pulp and cross-linking, due to the promoted heterogeneous nucleation which nevertheless fastens the crystallization but does not change the crystalline portion.^17,32^ TGA was carried out to investigate how the reaction influences the thermal degradation of the materials (Figure S4 and Table S2 in the Supporting Information). X-PBAT has lower onset and degradation temperatures compared to the neat matrix, due to the presence of low molecular weight chains resulting from β-scission induced by peroxide (Figure 2).^32^ The presence of pulp is reflected in an earlier onset of degradation in the biocomposites compared to that of PBAT, as expected after the addition of a lower thermostable component. However, PBAT protects pulp fibers from thermal degradation increasing their onset of about 30 °C. The effect of cross-linking in the biocomposite results in a slight improvement of the onset of P-PBAT, in line with the creation of a hybrid network.

### Sustainability Aspects

**Figure 7:**
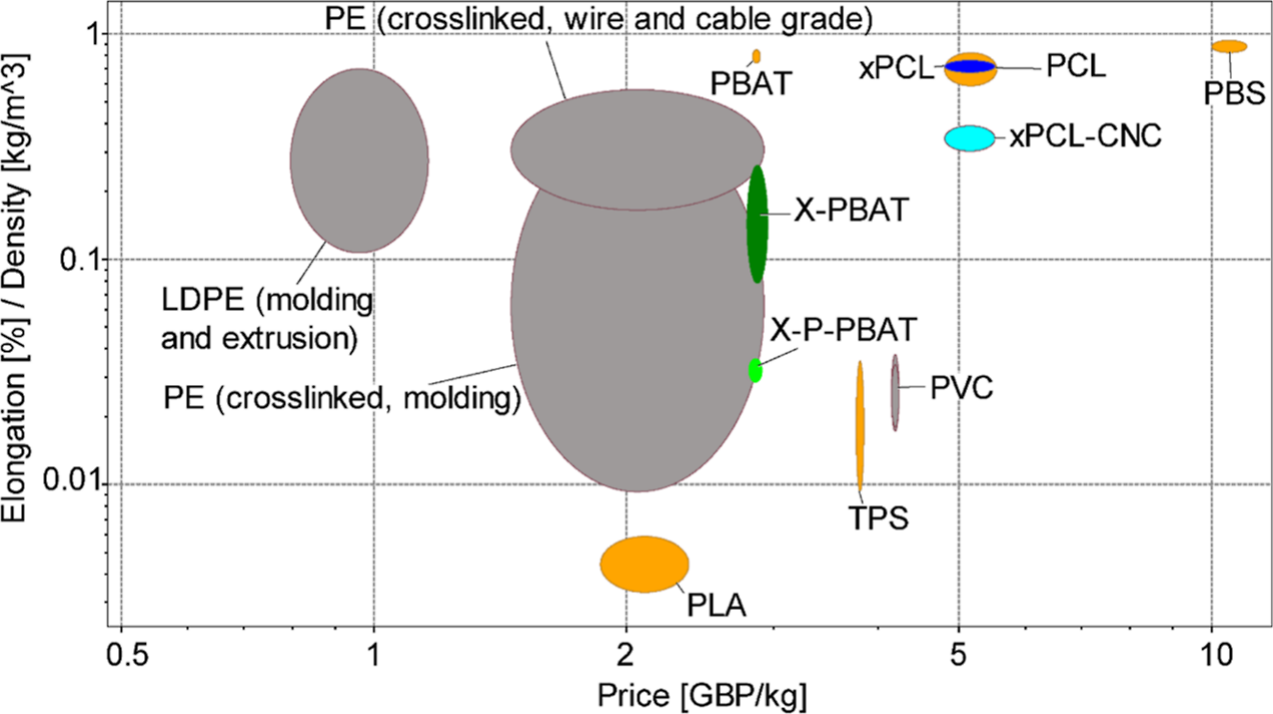
Ashby plot created with Granta EduPack software of specific elongation against the specific price of the cross-linked materials from this study in comparison with different polyethylene grades, polyvinyl chloride, and biodegradable/biobased polymers on the market (polybutylene succinate, polylactide, thermoplastic starch, and polycaprolactone). The plot also reports cross-linked PCL materials produced in our previous study.^18^ The prices of PBAT, pulp fibers, and PVC shrink films correspond to the grades purchased and used in this work (specifications in Materials section). The prices of PCL, PLA, PE and TPS are obtained from Granta EduPack database.

To evaluate whether the produced materials could replace the shrink films on the market, their mechanical properties and price were compared with commercial heat-shrinkable PVC wrap and cross-linked polyethylene, and with other biobased/biodegradable commercial polymers. The Ashby plot in Figure 7 reports the specific elongation as a function of the price of selected materials. Compared to the targeted PVC shrink wrap, the cross-linked PBAT and biocomposite are cheaper and have higher specific elongation, in addition to their biodegradability, nontoxicity and higher renewable content. The properties of X-PBAT and X-P-PBAT lie in the same range of cross-linked polyethylene. The reactive processing strategy adopted in this study is therefore economically viable to produce sustainable heat-shrinkable biocomposites that can replace fossil-based and nonbiodegradable packaging.

To further investigate the economical/environmental benefits of more valuable end-of-life options than incineration and landfilling, mechanical recycling and biodegradation of the materials have been evaluated, which are not taken into account in the Ashby economical assessment.

**Figure 8:**
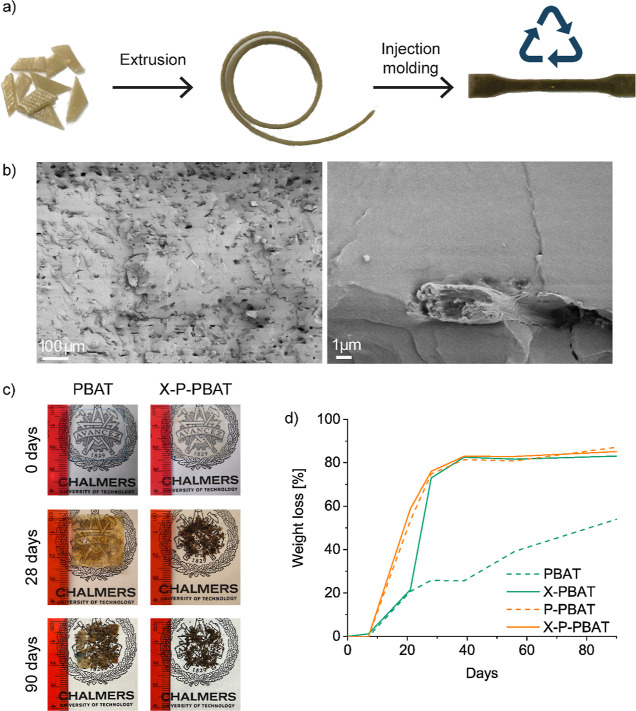
(a) Scheme of mechanical recycling procedure: the compression-molded films are shredded, extruded, and injection molded into tensile specimens. (b) Scanning electron microscopy of cryo-fractured surface of mechanically recycled X-P-PBAT at two different magnifications with scale bars of 100 and 1 μm. (c) Photographs of PBAT and X-P-PBAT films before and after 28 and 90 days of industrial composting. (d) Weight loss of the materials during 90 days of industrial composting. The logo in the photographs is used with permission of Chalmers University of Technology.

The mechanical recycling of the cross-linked materials was carried out by extrusion of shredded compression-molded films (Figure 8a). This test was a simulation of postindustrial waste recycling as it did not take into account the effects of consumers use of the films. The gel content of the cross-linked materials (≈55%) and their increased viscosity did not hinder the extrusion process, confirming mechanical recycling as a viable end-of-life option. The thermal stability of the materials was evaluated by TGA, which showed that the degradation was not affected by recycling (Figure S6 and Table S2 in the Supporting Information), indicating that the reprocessing does not lead to further significant chain scission or fiber degradation. The mechanical characterization performed by tensile tests revealed a slight stiffening (≈25% Young’s modulus increase) of the recycled biocomposite compared to X-P-PBAT (Table 2). The increase in the stiffness, not registered in the recycled X-PBAT, can be ascribed to further dispersion/fibrillation of the pulp fibers within the matrix achieved by reprocessing.^46^ This hypothesis found confirmation in the morphological analysis performed on the cryo-fractured recycled X-P-PBAT (Figure 8b), which showed individualized fibers, fewer fiber bundles, and fewer pull-outs. The cross-linked materials developed in this study can be therefore recycled, highlighting the advantage of their use as a replacement for PVC heat-shrinkable films. Indeed, PVC is difficult to mechanically recycle because its degradation generates chlorinated products that are toxic and corrosive to melt processing equipment.^3^

A further advantage of PBAT over other fossil-based polymers, such as polyethylene and PVC is its biodegradability. To assess whether the reactive melt processing and pulp fibers influence PBAT biodegradation, disintegration under composting conditions was carried out following the ISO 20200:2015 standard (Figures 8c and S7 in Supporting Information). The weight loss over 90 days of industrial composting shows that cross-linking increases PBAT disintegration rate, as a consequence of the larger polydispersity, as low molecular weight polyester chains are more readily available for microorganisms to biodegrade^47^ thus catalyzing the PBAT disintegration (Figure 8d). Similar results have been reported for PCL biodegradation, enhanced by radiation cross-linking.^48^ However, after 40 days, the biodegradation rate remarkably decreases. This result can be explained by the biodegradation path for synthetic polyesters, which progresses via hydrolytic degradation starting from the amorphous to the crystalline domains and continue with bacterial attack only when the molecular chains become oligomers.^11,49,50^ Pulp also increases the PBAT weight loss under composting, due to its faster biodegradation compared to the matrix,^51^ offering hydrophilic surfaces and not perfectly adherent interface with the matrix which promotes water and microorganisms penetration within the biocomposite bulk.^52,53^ After 90 days the weight loss of both P-PBAT and the cross-linked X-P-PBAT films is around 85% compared to 55% of neat PBAT, indicating a positive influence of the pulp fibers on the PBAT biodegradation.

## Conclusions

This work aimed to design circular biocomposites that could be applied as heat-shrinkable films and replace current fossil-based nonbiodegradable plastics, such as the currently used films in polyvinyl chloride or cross-linked polyethylene. The biocomposites were produced by reactive melt processing of biodegradable poly(butylene adipate-*co*-terephthalate) (PBAT) and renewable pulp fibers. Thanks to the radical reactions taking place during melt processing, the pulp fibers and the PBAT were cross-linked to form a hybrid insoluble 3D network in which all the fibers were irreversibly incorporated. The designed reactive melt processing led to a controlled gel content of ≈55 wt % in both the cross-linked PBAT and the biocomposite, which showed increased melt elasticity and creep resistance compared to the unreacted references. The cross-linked PBAT and biocomposite can shrink during creep above the matrix melting temperature. This entropy-driven phenomenon, triggered by temperature, indicated shape-memory of the cross-linked materials, which was demonstrated by the retention of their shape during four loading/heating cycles. Tensile tests indicated that pulp contributes to the stiffening of the biocomposite while cross-linking has the effect of limiting the embrittlement caused by the only addition of the pulp fibers. The morphological analysis highlighted that cross-linking led to an improved pulp dispersion and interaction with the matrix, explaining the regained deformability of the cross-linked biocomposites. To demonstrate the circularity, mechanical recycling, and industrial composting were performed on the materials. Despite the partial cross-linking of the systems, the materials could be reprocessed for mechanical recycling, with no significant impact on their thermal degradation and tensile properties. In simulated industrial composting of the films, cross-linking and the addition of pulp accelerated PBAT disintegration and increased its weight loss to 85 wt % after 90 days, against a 55 wt % loss measured for the neat matrix. The developed system can therefore be a sustainable cost-competitive alternative to currently used shrinkable polyvinyl chloride or cross-linked polyethylene, with the advantages of being recyclable, biodegradable, and partially renewable.

## Supporting Information Available

FTIR spectra of the pristine materials and Soxhlet extracted fractions; photos of cross-linked films before and after heat-shrinkage; shape-memory properties; DSC of the melt-processed materials; TGA of the melt-processed materials; thermal properties; DSC of the recycled materials; TGA of the recycled materials; photos of samples recovered from industrial composting (PDF)
