# Supplementary material for: Reusable, Recyclable, and Biodegradable Heat-Shrinkable Melt Cross-Linked Poly(butylene adipate-co-terephthalate)/Pulp Biocomposites for Polyvinyl Chloride Replacement
Source: ACS Sustain Chem Eng. 2024 Mar 15;12(13):5251–62. doi: 10.1021/acssuschemeng.4c00012 (PMC10988786; doi:10.1021/acssuschemeng.4c00012)
Supplement: Supplementary file 1 — sc4c00012_si_001.pdf [file sc4c00012_si_001.pdf]

Supporting Information

Reusable, recyclable and biodegradable heat-shrinkable melt crosslinked poly(butylene adipate-co-terephthalate)/pulp biocomposites for polyvinyl chloride replacement

Angelica Avella,[a] *Mathieu Salse,*[a,b] *Valentina Sessini,*[a,c] *Rosica Mincheva,*[d] *Giada Lo Re* [a]*

[a] Department of Industrial and Materials Science, Chalmers University of Technology, Rännvägen 2A, 41258 Gothenburg, Sweden

[b] Laboratoire MATEIS, Institut national des sciences appliquées Lyon, Bât. B. Pascal, Avenue Jean Capelle, 69621 Villeurbanne, France

[c] Department of Organic and Inorganic Chemistry, Institute of Chemical Research "Andrés M. del Río" (IQAR), Universidad de Alcalá, Campus Universitario, 28871 Alcalá de Henares, Madrid, Spain

[d] Laboratory of Polymeric and Composite Materials, University of Mons (UMons), 7000 Mons, Belgium

*Corresponding author: Giada Lo Re giadal@chalmers.se

This supplemental document contains 5 pages (S1-S5), Figures S1-S7, and Tables S1-S2.

Contents:

Figure S1: FTIR spectra of the pristine materials and Soxhlet extracted fractions………S2

Figure S2: Photos of crosslinked films before and after heat-shrinkage…………………S2

Table S1: Shape-memory properties……………………………………………………...S2

Figure S3: DSC of the melt-processed materials…………………………………………S3

Figure S4: TGA of the melt-processed materials…………………………………………S3

Table S2: Thermal properties………………………………………………………………S3

Figure S5: DSC of the recycled materials…………………………………………………S4

Figure S6: TGA of the recycled materials………………………………………………...S4

Figure S7: Photos of samples recovered from industrial composting…………………….S5

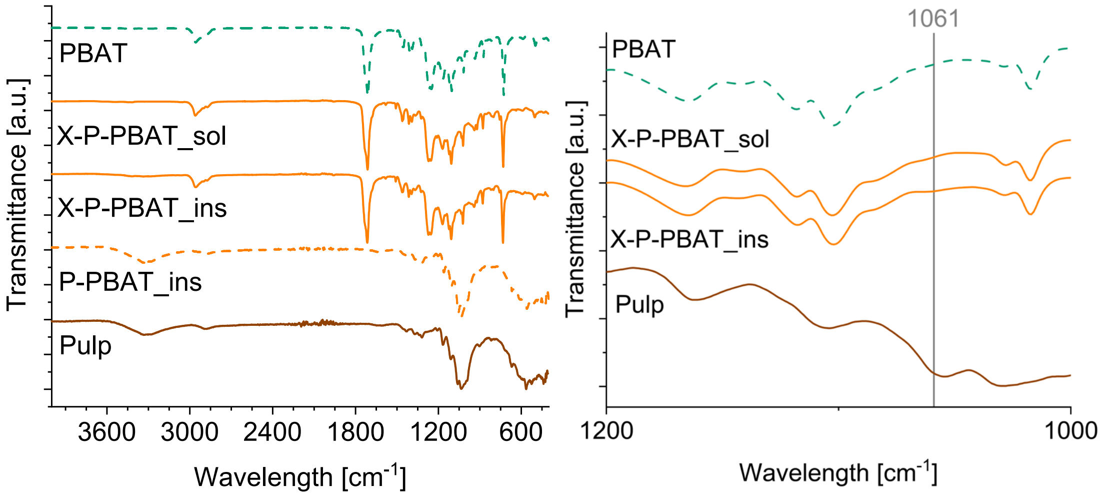

Figure S1. Attenuated Total Reflectance Fourier-Transform Infrared Spectroscopy in transmission of neat PBAT and pulp, soluble fraction of X-P-PBAT and insoluble fractions of X-P-PBAT and P-PBAT, with an inset around 1061 cm^{-1}.

a) b)

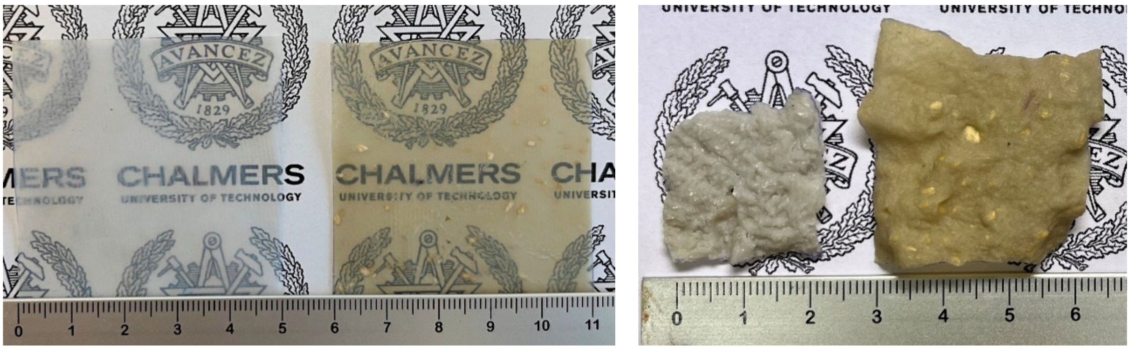

Figure S2. Photographs of X-PBAT (left) and X-P-PBAT (right) films before (a) and after (b) being exposed to 160 °C in an oven for 5 min. The logo in the photographs is used with permission of Chalmers University of Technology.

Table S1. The maximum strain (ε_m), the strain after the stress was removed (ε_u) the strain in the recovered state (ε_p), shape-recovery (R_r) and the shape-fixity (R_f) ratios for X-P-PBAT and X-PBAT during the thermo-mechanical cycles.

Material	*Cycle*	ε_m *[%]*	ε_u *[%]*	ε_p *[%]*	*Rf*	*Rr*
X-P-PBAT	1	35	35	3	100	91
	2	34	34	2	100	94
	3	35	35	2	100	94
	4	34	34	2	100	94
X-PBAT	1	84	83	5	99	94
	2	85	84	5	99	94
	3	112	111	7	99	94
	4	97	96	6	99	94

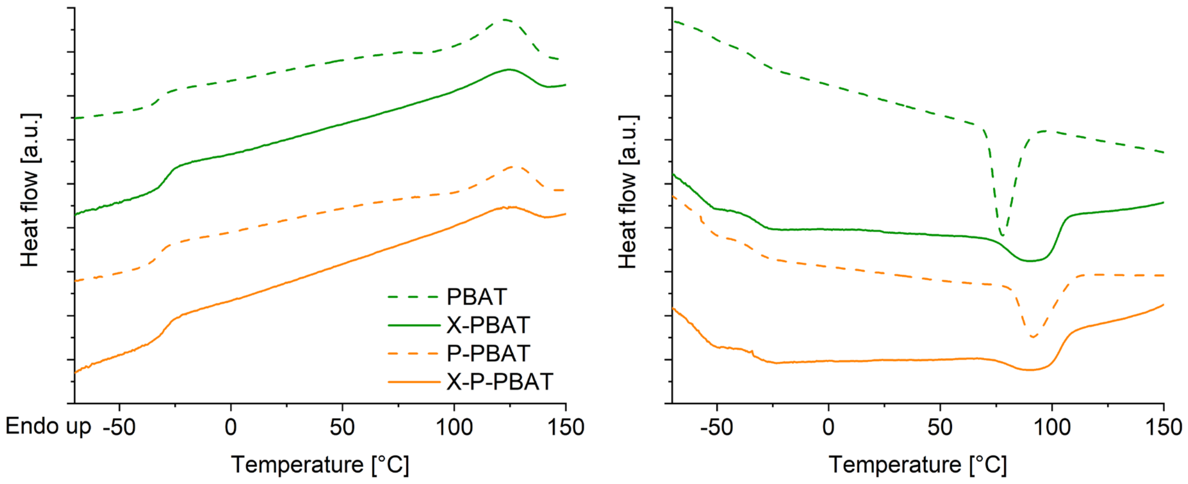

Figure S3. Differential Scanning Calorimetry (DSC) curves recorded during the second heating (left) and cooling (right).

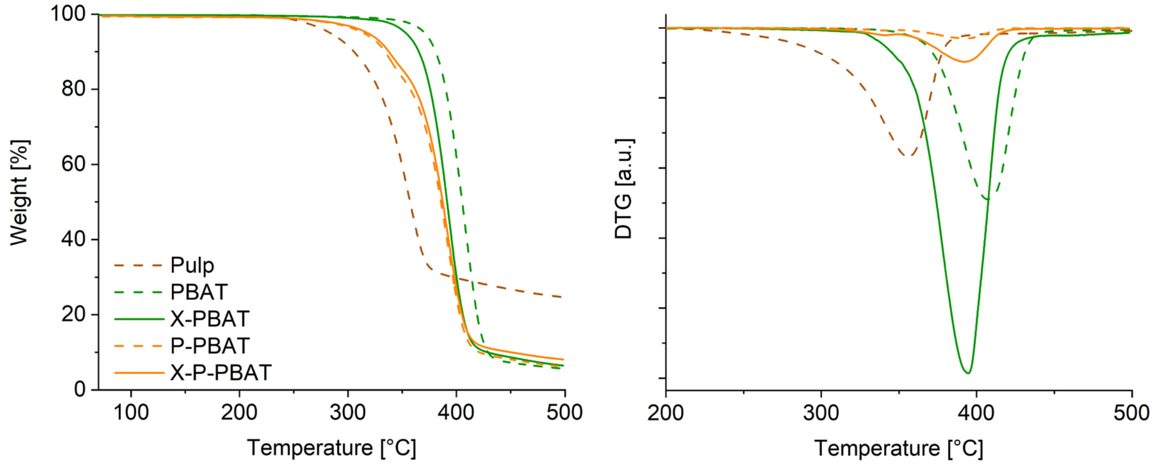

Figure S4. Thermogravimetric analysis (TGA) under nitrogen (left) and first derivative curves (right).

Table S2. Thermal properties obtained from DSC and TGA: melting temperature (T_m), melting enthalpy (ΔH_m) and crystallinity (χ_c); crystallization temperature (T_c); temperature at 5% weight loss ($T_{5\%}$); the peak temperatures of degradation (T_{d1}, T_{d2}); char residue at 500 °C.

Material	T_g *[°C]*	T_m *[°C]*	T_c *[°C]*	ΔH_m *[J/g]*	χ_c *[%]*	$T_{5\%}$ *[°C]*	T_{d1} *[°C]*	T_{d2} *[°C]*	*Char [%]*
Pulp	-	-	-	-	-	284	356	-	25
PBAT	-33	123	78	13	11	361	-	407	6
X-PBAT	-29	125	90	9	8	353	-	394	6
P-PBAT	-32	128	92	8	8	314	339	393	6
X-P-PBAT	-30	124	90	7	7	318	340	392	8
Re-X-PBAT	-30	123	101	6	5	355	-	394	8
Re-X-P-PBAT	-29	122	99	6	6	314	339	394	8

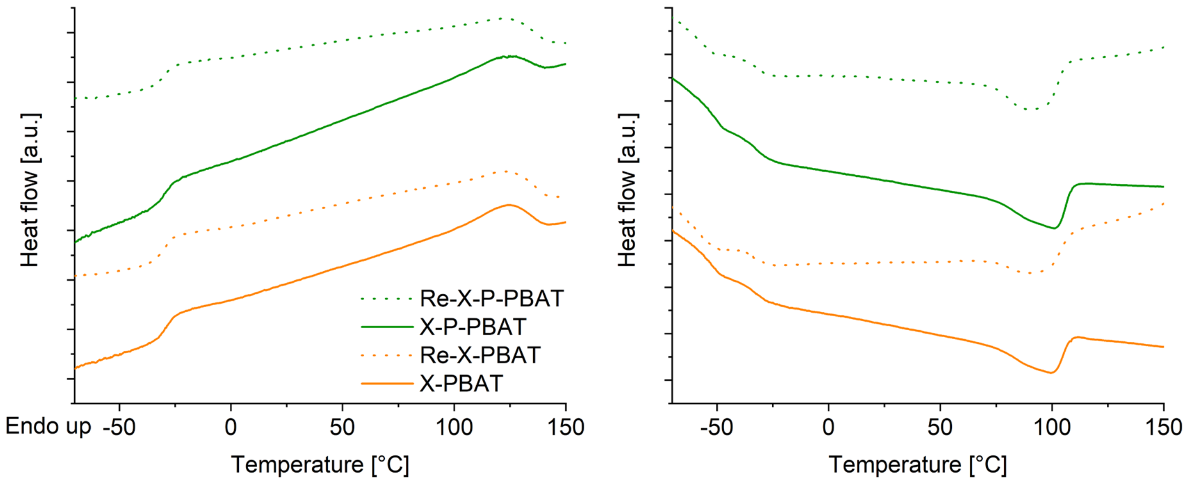

Figure S5. DSC curves of the mechanically recycled materials compared to the pristine ones recorded during the second heating (left) and cooling (right).

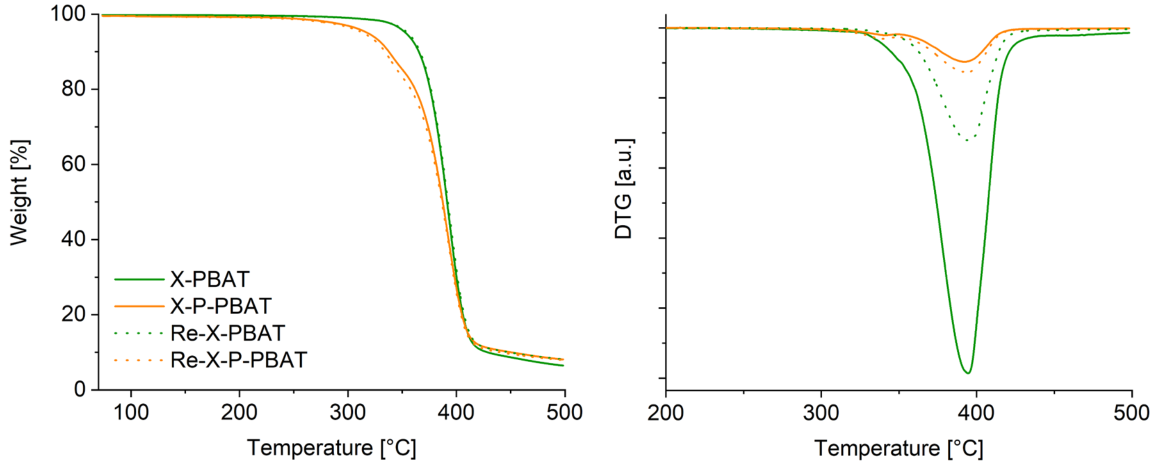

Figure S6. TGA under nitrogen (left) and first derivative curves (right) of the mechanically recycled materials compared to the pristine ones.

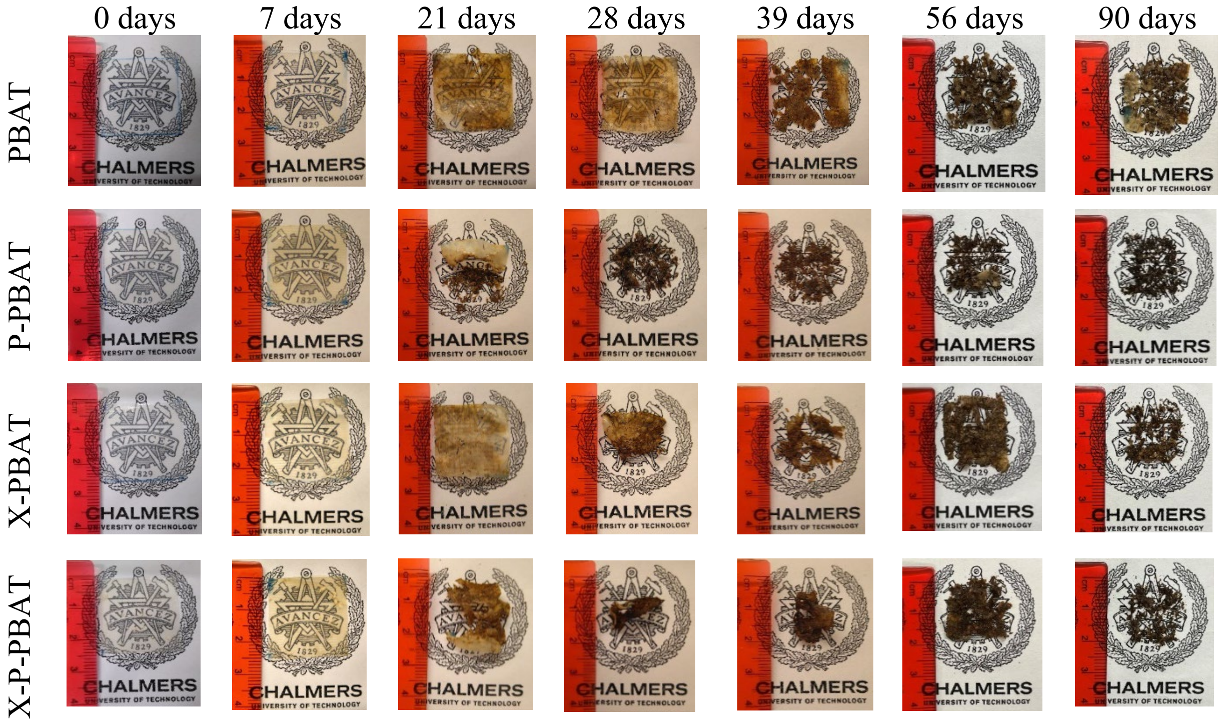

Figure S7. Photographs of the materials' films recovered during 90 days of industrial composting. The logo in the photographs is used with permission of Chalmers University of Technology.
